# Small‐Bowel Impaction Caused by Mechanical Interlocking Between Duodenal Metal and Biliary Plastic Stents

**DOI:** 10.1002/deo2.70362

**Published:** 2026-06-17

**Authors:** Shinsuke Otagiri, Ryo Sugiura, Masataka Wada, Asako Nomura, Hiroki Egami, Kento Wakabayashi, Kenta Fujihata, Takehiko Katsurada, Satoshi Hirano, Naoya Sakamoto

**Affiliations:** ^1^ Department of Gastroenterology and Hepatology Hokkaido University Hospital Sapporo Japan; ^2^ Department of Gastroenterological Surgery II Hokkaido University Hospital Sapporo Japan; ^3^ Department of Surgical Pathology Hokkaido University Hospital Sapporo Japan

**Keywords:** double‐balloon enteroscopy, duodenal stent, pancreatic cancer, small‐bowel impaction, stent migration

## Abstract

Duodenal self‐expandable metal stents (SEMS) are widely used to treat malignant gastric outlet obstruction (GOO). However, stent migration is a well‐known complication. We report a rare case of small‐bowel impaction caused by the migration of a duodenal SEMS with a biliary plastic stent (PS). A 78‐year‐old woman with pancreatic head cancer underwent biliary PS and duodenal SEMS placement for obstructive jaundice and GOO. At 4 months after initiating neoadjuvant chemotherapy, computed tomography revealed tumor shrinkage and improvement in duodenal stenosis; however, the SEMS and PS had migrated to the ileum. Double‐balloon enteroscopy was attempted; however, stent removal was unsuccessful because of mechanical interlocking and impaction. Therefore, a partial ileal resection was performed. Histopathological examination revealed transmural infiltration of inflammatory cells and abscess formation. If a duodenal SEMS migrates with a biliary PS, mechanical interlocking may occur, resulting in small‐bowel impaction. Clinicians should be aware of this rare complication and should consider careful follow‐up and timely intervention if stent migration is detected.

**Trial Registration**: N/A

## 1 Introduction

Duodenal self‐expandable metal stents (SEMSs) are widely used to treat malignant gastric outlet obstruction (GOO), including duodenal obstruction caused by pancreatic or periampullary malignancies. Although stent migration is a known complication [1], it often traverses the gastrointestinal tract without causing symptoms. However, in some cases, the migrated stents may be retained in the small intestine, leading to severe complications [2]. Furthermore, multiple stentings may result in unexpected mechanical interlocking, preventing spontaneous passage. We report a rare case of small‐bowel impaction caused by mechanical interlocking between a migrated duodenal SEMS and a biliary plastic stent (PS) following prolonged neoadjuvant chemotherapy (NAC) for pancreatic head cancer.

## 2 Case Report

**FIGURE 1:**
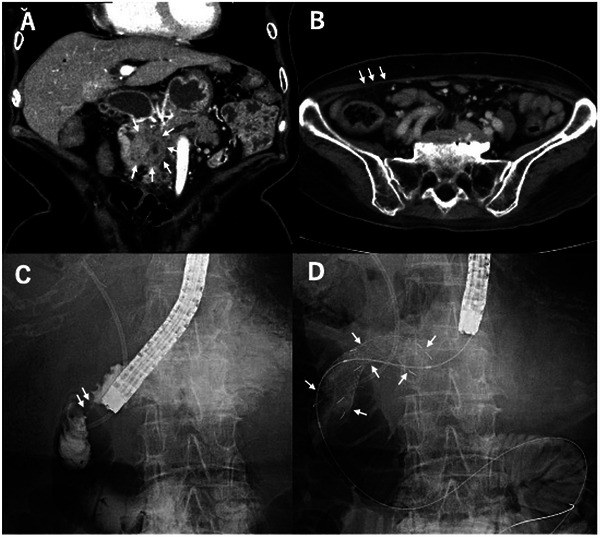
(A, B) Computed tomography (CT) image obtained before chemotherapy initiation. (A) Coronal CT image showing a 40‐mm mass in the head of the pancreas (white arrow) with upstream pancreatic duct dilatation and duodenal stenosis secondary to tumor invasion. (B) Axial CT image of the right lower abdomen showing no evidence of peritoneal dissemination, adhesions, or structural abnormalities at the site of subsequent stent impaction (white arrow). (C, D) Fluoroscopic images during placement of a duodenal stent. (C) Localized malignant stenosis at the duodenal bulb (white arrow). (D) Placement of a self‐expandable metallic stent (white arrow).

A 78‐year‐old woman with a history of distal gastrectomy (Billroth I reconstruction) for a gastric tumor 25 years prior was admitted to a previous hospital with obstructive jaundice. Contrast‐enhanced computed tomography (CT) revealed a 40‐mm mass in the pancreatic head with upstream pancreatic duct dilatation and duodenal stenosis (Figure 1A). A 7‐Fr, 9‐cm biliary PS (QuickPlace V; Olympus Medical Systems Corp., Tokyo, Japan) was placed for biliary drainage.

The patient was then referred to our hospital for further evaluation. Endoscopic ultrasound‐guided tissue biopsy of the pancreatic tumor confirmed the presence of an adenocarcinoma. CT revealed no distant metastasis or peritoneal dissemination. In addition, no structural abnormalities were identified in the right lower abdomen at the site of subsequent stent impaction (Figure 1B). No tumor contact with the celiac axis or the superior mesenteric artery was observed. Serum carbohydrate antigen 19‐9 (CA19‐9) levels were elevated (728.7 IU/mL). Therefore, the patient was diagnosed with biological borderline resectable pancreatic head cancer based on the elevated CA19‐9 level (>500 IU/mL) in accordance with the International Consensus Definition and Criteria for Borderline Resectable Pancreatic Ductal Adenocarcinoma [3], despite the absence of clear anatomical borderline features on imaging. The tumor was classified as clinical stage IB (cT2N0M0).

Radical surgical resection was planned after NAC. As the patient initially had no symptoms of GOO, NAC with gemcitabine plus nab‐paclitaxel was initiated. However, obstructive symptoms soon developed, and a duodenal uncovered SEMS (UC‐SEMS) (22 × 80 mm, Gentry; Japan Lifeline Co., Ltd., Tokyo, Japan) was placed 3 days after the first administration of NAC. The stent extended from the prepyloric region to the descending duodenum, crossing the papilla with its distal end positioned beyond the biliary PS (Figure 1C,D). Endoscopic stent placement was selected to achieve rapid symptom relief while minimizing disruption of chemotherapy.

**FIGURE 2:**
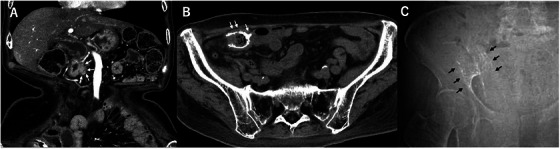
(A, B) Computed tomography (CT) images at 4 months after initiating chemotherapy. (A) The pancreatic tumor (white arrow) shows marked shrinkage, and the duodenal stenosis has improved. (B) The migrated duodenal metallic and biliary plastic stents are in the right lower abdomen (white arrow). (C) Abdominal radiograph obtained 2 weeks after CT shows no change in stent position (black arrow).

**FIGURE 3:**
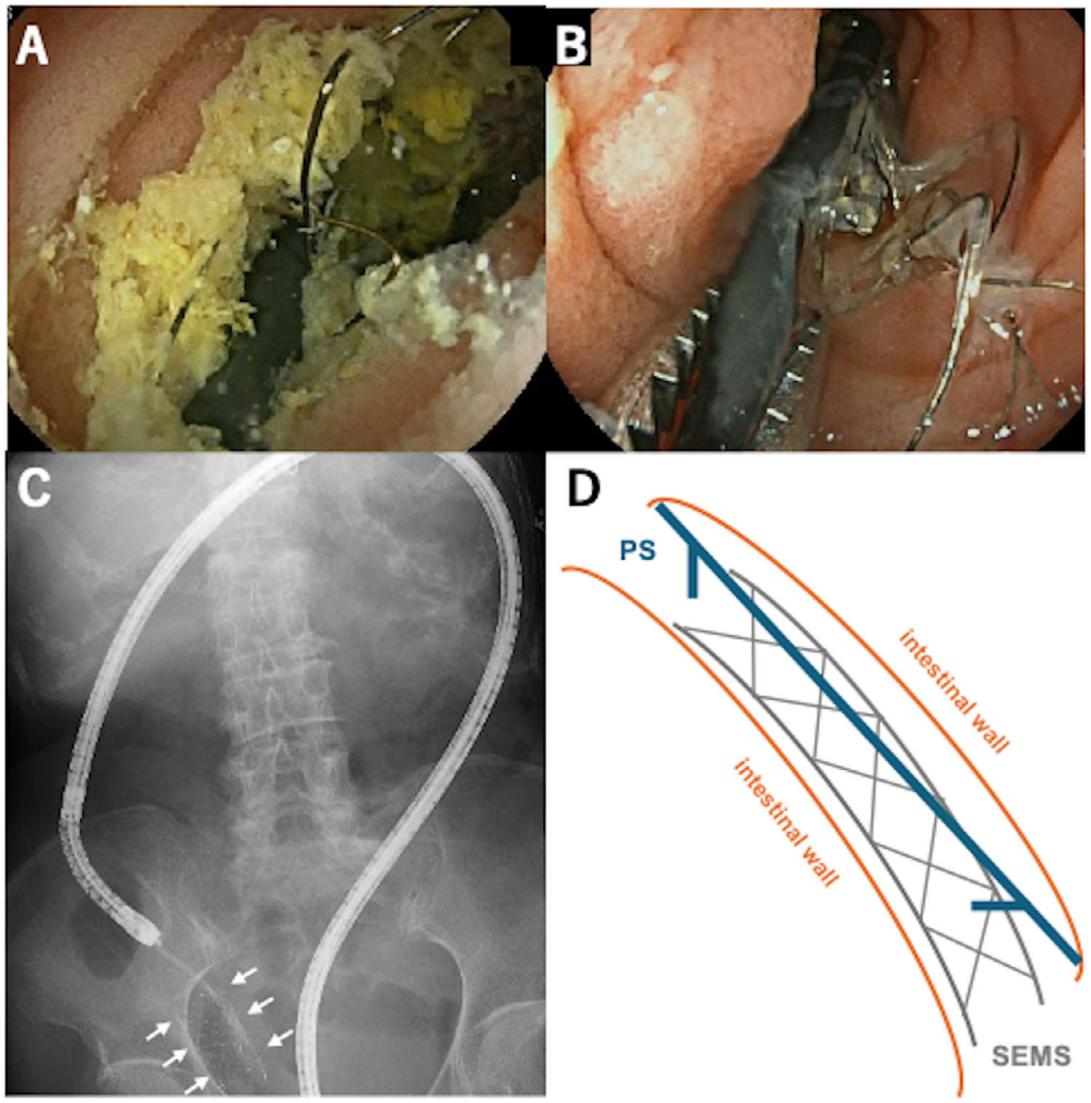
(A, B) Endoscopic images obtained during transanal double‐balloon enteroscopy. (A) The duodenal self‐expandable metal stent (SEMS) and the biliary plastic stent (PS) are mechanically interlocked and impacted in the ileum. (B) Attempted retrieval of the biliary PS using grasping forceps (alligator type). (C) A fluoroscopic image obtained during an endoscopic procedure. Traction of the biliary PS is unsuccessful because of mechanical interlocking with the duodenal SEMS. (D) Schematic illustration of mechanical interlocking between a duodenal SEMS and a biliary PS. The distal flap of the biliary PS was engaged with an SEMS mesh, preventing disengagement. As a result, the two stents behave as a single rigid unit, leading to small‐bowel impaction.

Two months after initiation, a follow‐up CT revealed tumor shrinkage, and the duodenal UC‐SEMS and biliary PS remained in the appropriate position. Four months after initiating NAC, CA19‐9 levels markedly decreased to 8.8 IU/mL, and CT revealed further tumor shrinkage and improvement in duodenal stenosis (Figure 2A). However, the duodenal UC‐SEMS and biliary PS had migrated to the right lower abdomen, and their configuration suggested migration into the ileum (Figure 2B). The patient remained asymptomatic. An abdominal radiograph obtained after 2 weeks showed no interval change in the position of the stents (Figure 2C). As no interval movement was observed and intestinal perforation was possible, transanal double‐balloon enteroscopy was performed. Endoscopic examination revealed that the UC‐SEMS and biliary PS were lodged in the ileum (Figure 3A). Removal of the biliary PS was attempted using endoscopic grasping forceps (alligator type) (Figure 3B); however, traction failed because the PS was firmly fixed within the UC‐SEMS mesh, possibly because of the engagement of its distal flap with the mesh (Figure 3C,D). Fluoroscopic imaging confirmed that the stents remained impacted and immobile at the same location, with no apparent mobility in the surrounding intestinal segment (Figure 3C). The use of a snare was not feasible because of the lack of an adequate working space. In addition, removal of the UC‐SEMS was unsuccessful because of its fixation to the intestinal wall. Because endoscopic retrieval was impossible, surgical management was planned. Although radical resection for pancreatic cancer had initially been planned following NAC, the patient's performance status deteriorated from 0 to 2, accompanied by a decrease in serum albumin levels from 3.5 to 2.1 g/dL during NAC. Considering the poor general condition, curative resection was deferred, and only intestinal resection was performed.

**FIGURE 4:**
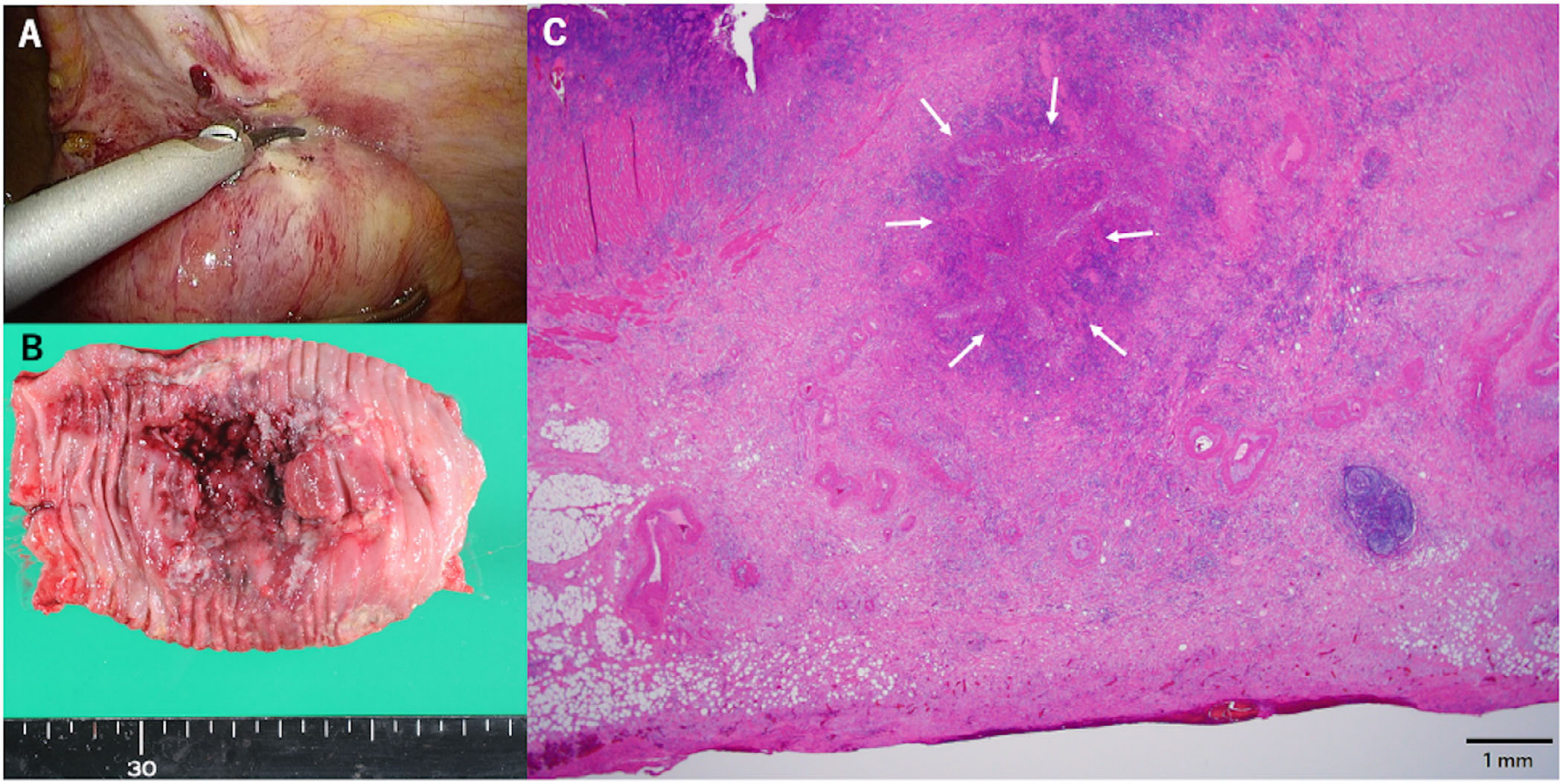
(A) Intraoperative findings show inflammatory adhesion of the ileum containing the migrated stent to the abdominal wall. (B) Gross pathological specimen shows ulcer formation at the site of metallic stent lodging. (C) Histopathological findings on the serosal side of the resected specimen (hematoxylin and eosin staining, ×1.25) demonstrate transmural inflammatory cell infiltration and abscess formation (white arrow).

The ileum was partially resected. Intraoperatively, the ileum containing the migrated stents adhered to the abdominal wall because of inflammatory changes (Figure 4A). The impaction site was located approximately 15 cm proximal to the ileocecal valve, which was consistent with the location identified on preoperative CT. No macroscopic evidence of peritoneal dissemination was observed. The resected specimen showed ulcer formation at the site where the duodenal UC‐SEMS was lodged in direct contact with the UC‐SEMS, rather than the biliary PS (Figure 4B). Histopathological examination revealed transmural inflammatory cell infiltration, with abscess formation in the affected intestinal wall (Figure 4C), and malignant cells were not detected.

The patient recovered without any major postoperative complications. To date, no recurrent GOO or biliary obstruction has been observed. Maintenance therapy with S‐1 was initiated, and radical resection was planned following an improvement in the general condition.

## 3 Discussion

Successful placement of a duodenal SEMS relieves obstructive symptoms and has a relatively low complication rate [4]. The migration rate of the UC‐SEMSs ranges from 0% to 11% [5]. Migration may be facilitated by reduced tissue anchoring and stent‐related mechanical factors, including the axial expansion force and structural characteristics of the stent [6]. Migrated stents often pass spontaneously; however, in some cases, they may lodge in the small intestine, prompting intervention [5].

Mechanical interlocking between the duodenal SEMS and biliary PS, resulting in small‐bowel impaction, has not been previously reported. Previous studies on stent placement for malignant biliary obstruction have demonstrated that the placement of an additional PS can reduce the migration of fully covered SEMS by providing an anchoring effect [7, 8], suggesting that interactions between the stents may influence their mechanical behavior. Presently, the PS may have functioned as a mechanical anchor.

The exact timing of the mechanical interlocking remains uncertain, as it may have occurred either at the time of UC‐SEMS placement or after migration into the small intestine. Regardless of the timing, interlocking likely increased the risk of impaction by causing the stents to behave as a single rigid unit and impairing spontaneous passage, possibly because of the engagement of the distal flap of the straight‐type PS with the SEMS mesh (Figure 3D). In contrast to a straight‐type PS, a pigtail‐type biliary PS is less likely to traverse the SEMS mesh and cause mechanical interlocking. Although impaction with SEMS alone has been reported [5], the presence of a straight‐type PS may further increase this risk.

Intraoperatively, the ileum containing the migrated stents was adhered to the abdominal wall. These findings suggest that prolonged impaction of the stents induced severe localized inflammation extending from the mucosa to the deeper layers of the intestinal wall. This inflammatory process may have progressed to focal transmural injury and microperforation, ultimately resulting in adhesion to the abdominal wall [9]. Although no definite perforation was identified histologically, transmural inflammation with focal wall destruction (Figure 4C) supported bowel fragility and was consistent with possible microperforation. Alternative explanations, such as preexisting adhesions related to previous abdominal surgery or occult peritoneal dissemination, were considered; however, neither intraoperative nor pathological findings supported these possibilities.

Early endoscopic intervention may be beneficial in select cases of stent migration. Successful endoscopic retrieval of the migrated duodenal SEMS from the ileum using double‐balloon enteroscopy has been reported [1]. Retrospectively, earlier intervention may have been considered. However, immediate endoscopic removal was challenging because the patient was asymptomatic and spontaneous passage of the stents was initially expected.

Tumor shrinkage following anticancer therapy may lead to stent migration by reducing tumor‐related anchoring. The migration of biliary and esophageal SEMSs has been reported [10]; however, the migration of duodenal SEMS following NAC has not been previously described.

Overall, if a duodenal SEMS migrates with a biliary PS, mechanical interlocking may occur, resulting in small‐bowel impaction that requires surgical intervention. Careful follow‐up imaging is important, and early endoscopic retrieval may be considered if the migrated stents fail to progress.

## Author Contributions

**Shinsuke Otagiri**: Conceptualization, writing the original draft, and patient management. **Ryo Sugiura**: Patient management, writing, review, and editing. **Masataka Wada**: Surgical management. **Asako Nomura**: Patient management. **Hiroki Egami**: Patient management. **Kento Wakabayashi**: Pathological evaluation. **Kenta Fujihata**: Patient management. **Takehiko Katsurada**: Supervision. **Satoshi Hirano**: Supervision. **Naoya Sakamoto**: Supervision. All the authors approved the final version of the manuscript.

## Funding

The authors have nothing to report.

## Ethics Statement

Approval of the Research Protocol by An Institutional Review Board: N/A.

## Consent

Written informed consent was obtained from the patient for the publication of this case report and any accompanying images.

## Conflicts of Interest

The authors declare no conflicts of interest.
